# Extracellular volume is an independent predictor of arrhythmic burden in dilated cardiomyopathy

**DOI:** 10.1038/s41598-021-03452-z

**Published:** 2021-12-14

**Authors:** Pawel P. Rubiś, Ewa M. Dziewięcka, Paweł Banyś, Małgorzata Urbańczyk-Zawadzka, Maciej Krupiński, Małgorzata Mielnik, Jacek Łach, Andrzej Ząbek, Sylwia Wiśniowska-Śmiałek, Piotr Podolec, Aleksandra Karabinowska, Katarzyna Holcman, Ann C. Garlitski

**Affiliations:** 1 Department of Cardiac and Vascular Diseases, Jagiellonian University Medical College, John Paul II Hospital, Pradnicka Street 80, 31-202 Krakow, Poland; 2 Department of Radiology, John Paul II Hospital, Pradnicka Street 80, 31-202 Krakow, Poland; 3 Department of Electrocardiology, John Paul II Hospital, Pradnicka Street 80, 31-202 Krakow, Poland; 4 Tufts Medical Center Boston, 800 Washington Street, Boston, MA 02111 USA

**Keywords:** Cardiology, Cardiovascular diseases

## Abstract

The current stratification of arrhythmic risk in dilated cardiomyopathy (DCM) is sub-optimal. Cardiac fibrosis is involved in the pathology of arrhythmias; however, the relationship between cardiovascular magnetic resonance (CMR) derived extracellular volume (ECV) and arrhythmic burden (AB) in DCM is unknown. This study sought to evaluate the presence and extent of replacement and interstitial fibrosis in DCM and to compare the degree of fibrosis between DCM patients with and without AB. This is a prospective, single-center, observational study. Between May 2019 and September 2020, 102 DCM patients underwent CMR T1 mapping. 99 DCM patients (88 male, mean age 45.2 ± 11.8 years, mean EF 29.7 ± 10%) composed study population. AB was defined as the presence of VT or a high burden of PVCs. There were 41 (41.4%) patients with AB and 58 (58.6%) without AB. Replacement fibrosis was assessed with late gadolinium enhancement (LGE), whereas interstitial fibrosis with ECV. Overall, LGE was identified in 41% of patients. There was a similar distribution of LGE (without AB 50% vs. with AB 53.7%; *p* = 0.8) and LGE extent (without AB 4.36 ± 5.77% vs. with AB 4.68 ± 3.98%; *p* = 0.27) in both groups. ECV at nearly all myocardial segments and a global ECV were higher in patients with AB (global ECV: 27.9 ± 4.9 vs. 30.3 ± 4.2; *p* < 0.02). Only indexed left ventricular end-diastolic diameter (HR 1.1, 95%CI 1.0–1.2; *p* < 0.02) and global ECV (HR 1.12, 95%CI 1.0–1.25; *p* < 0.02) were independently associated with AB. The global ECV cut-off value of 31.05% differentiated both groups (AUC 0.713; 95%CI 0.598–0.827; *p* < 0.001). Neither qualitative nor quantitative LGE-based assessment of replacement fibrosis allowed for the stratification of DCM patients into low or high AB. Interstitial fibrosis, expressed as ECV, was an independent predictor of AB in DCM. Incorporation of CMR parametric indices into decision-making processes may improve arrhythmic risk stratification in DCM.

## Introduction

Ventricular arrhythmias, including premature ventricular contractions (PVCs) and non-sustained ventricular tachycardia (NSVT), occur in 40% of patients with dilated cardiomyopathy (DCM)^1^. The bulk of evidence indicates that runs of NSVT and frequent PVCs, defined as arrhythmic burden (AB), lead to an increased sudden cardiac death (SCD) risk in DCM patients^2^. Susceptibility to ventricular arrhythmias in DCM relies on the combined presence of an anatomic substrate (i.e. genetic or acquired abnormalities in the electrical or mechanical properties of the heart) and triggering mechanisms. Profound cardiac remodeling and fibrosis provide ample substrate for the initiation of ventricular arrhythmias.

Cardiac fibrosis is typically observed in 40–60% of DCM patients^3,4^. Broadly, two types of fibrosis have been identified. Replacement or scarring fibrosis develops as a consequence of local myocytes death and serves to preserve the integrity and function of the heart after injury^5^. DCM is typically characterized with wide-spread interstitial fibrosis given that the need for cardiac repair is minimal and, ultimately, fibrosis is a maladaptive event^5^.

The preferred contemporary method of assessment of cardiac fibrosis is cardiovascular magnetic resonance (CMR)^6,7^. Identification of late gadolinium enhancement (LGE) allows for the detection of areas of replacement fibrosis, which are relatively large and focal^6,7^. However, LGE cannot be used to detect and quantify interstitial fibrosis^5–7^. T1 mapping has emerged as a robust technique for the quantification of interstitial fibrosis by measuring T1 times and extracellular volume (ECV)^5–8^. In fibrotic hearts, both native T1 time and ECV increase, whereas post-contrast T1 time decreases^6–8^. T1 times and ECV have been validated against biopsy-determined collagen volume fraction^9–11^.

Despite evidence suggesting that cardiac fibrosis is the crucial parameter influencing arrhythmic risk, current guidelines do not take fibrosis into account^12–14^. Studies on cardiac fibrosis, utilizing either LGE-based or T1-parametric assessment showed associations between fibrosis and clinically-relevant outcomes, including arrhythmic risk^15–17^. However, the relationship between comprehensive assessment of interstitial fibrosis, by means of ECV, and AB in DCM has not been established. The primary hypothesis underlying the study was that patients with high AB would have an increased ECV, suggesting a higher amount of interstitial fibrosis. Therefore, we compared the degree of interstitial fibrosis, expressed as ECV, between DCM patients with high and low AB.

## Results

### Baseline characteristics

Out of 102 initially recruited patients, 3 subjects had incomplete data such that the final study population consisted of 99 DCM patients. Eighty-eight (88.9%) patients were male, and the mean age was 45.2 ± 11.8 years. Patients were divided into those with (n = 41, 41.4%) and without (n = 58, 58.4%) AB. The comparison of the baseline demographic, clinical, laboratory, echocardiographic parameters, and therapies between the two groups is presented in Table 1. Patients with an AB had a larger indexed LV end-diastolic diameter (LVEDd; 32.6 ± 5.1 vs. 30.6 ± 4.2 mm; *p* < 0.05), whereas all other parameters were similarly distributed. No gender-based differences were identified.

**Table 1:** Baseline characteristic. Comparison of DCM patients with and without AB.

Parameter	without AB (n = 58)	with AB (n = 41)	*p* value
**Demographics**			
Age (year)	44.52 ± 11.37	45.64 ± 12.58	0.64
Sex—male (n, %)	54 (93.1%)	34 (82.9)	0.11
BMI (kg/m^2^)	28.89 ± 5.72	28.08 ± 5.79	0.54
NYHA class	1.76 ± 0.62	1.87 ± 0.65	0.57
SBP (mmHg)	122.9 ± 19.7	116 ± 18.8	0.07
DBP (mmHg)	78.4 ± 14.1	75.2 ± 13	0.08
6-MWT—distance (m)	447.1 ± 93.6	446.1 ± 93.9	0.96
6-MWT—Borg scale	2.11 ± 1.78	2.15 ± 1.61	0.52
**Medical history**			
HF symptoms duration (month)	14.73 ± 22.67	19.12 ± 24.84	0.25
Urgent HF hospitalisation within last 12 months (n, %)	40 (69.0)	24 (58.5)	0.29
Diabetes mellitus (n, %)	9 (15.5)	6 (14.6)	0.90
Hypercholesterolemia (n, %)	39 (67.2)	22 (53.7)	0.17
Hypertension (n, %)	16 (27.6)	5 (12.2)	0.07
Atrial fibrillation (n, %)	18 (31.0)	9 (22.0)	0.32
History of smoking (n, %)	27 (46.5)	24 (61.5)	0.34
**Echocardiography**			
LVEDd/BSA (mm/m^2^)	30.59 ± 4.22	32.59 ± 5.15	**0.046**
IVS (mm)	9.95 ± 1.80	10.11 ± 2.77	0.96
LVEF (%)	29.86 ± 10.69	29.11 ± 9.18	0.72
RVd/BSA (mm/m^2^)	19.63 ± 3.41	19.43 ± 3.4	0.78
TAPSE (mm)	19.41 ± 4.27	19.73 ± 3.84	0.70
LAVI (ml/m^2^)	55.34 ± 24.15	50.13 ± 21.68	0.53
E/e’	10.15 ± 5.59	10.65 ± 6.30	0.99
MR (n, %)	18 (31.0)	16 (39.0)	0.41
TR (n, %)	8 (13.8)	2 (4.9)	0.15
TRV (m/s)	2.57 ± 1.34	2.37 ± 1.45	0.34
**Laboratory**			
Hb (g/dl)	14.78 ± 1.62	14.79 ± 1.29	0.97
Hct (%)	43.48 ± 4.61	43.25 ± 3.73	0.84
Creatinine (umol/l)	95.79 ± 41.12	86.39 ± 19.69	0.09
Potassium (mmol/l)	4.66 ± 0.36	4.57 ± 0.33	0.30
hsCRP (mg/dl)	3.72 ± 8.12	3.54 ± 3.93	0.31
hsTnT (ng/ml)	0.0133 ± 0.012	0.045 ± 0.176	0.56
NT-proBNP (pg/ml)	998.6 ± 1480.4	1275.2 ± 1481.7	0.27
**Medications**			
BB (n, %)	58 (100.0)	41 (100.0)	1.00
ARNI/ACEi (n, %)	58 (100.0)	40 (97.6)	0.23
ARNI (n, %)	32 (55.2)	25 (61.0)	
ACEi (n, %)	26 (44.8)	16 (39.0)	
MRA (n, %)	55 (94.8)	40 (97.6)	0.50
Loop diuretics (n, %)	45 (77.6)	27 (65.9)	0.20
Loop diuretics daily dosage (mg/d)	42.4 ± 46.1	35.72 ± 79.54	0.07
OAC (n, %)	19 (32.7)	9 (22.0)	0.32

Values are mean ± SD or n (%).

*AB* arrhythmic burden, *BMI *body mass index, *HF* heart failure, *NYHA* New York Heart Association class, *SBP/DBP* systolic/diastolic blood pressure, *6-MWT* 6-min walk test, *LVEDd* left ventricle end-diastolic diameter, *BSA* body surface area, *LVEF* left ventricle ejection fraction, *RVd* right ventricle basal diameter from apical 4-chamber view, *TAPSE* tricuspid annular plane systolic excursion, *LAVI* left atria volume indexed, *MR/TR* moderate or severe mitral/tricuspid regurgitation, *TRV* TR peak velocity, *Hb* haemoglobin, *Hct* haematocrit, *hsCRP* high-sensitive C-reactive protein, *hsTnT* high-sensitive troponin T, *NT-proBNP* N-terminal pro b-type natriuretic peptide, *BB* beta-blocker, *ARNI* angiotensin receptor-neprilysin inhibitor, *ACEI* angiotensin-converting-enzyme inhibitor, *MRA* mineralocorticoid receptor antagonist, *OAC* (VKA and non-VKA) oral anticoagulants.

### Analyses of Holter and CMR parameters

**Figure 1:**
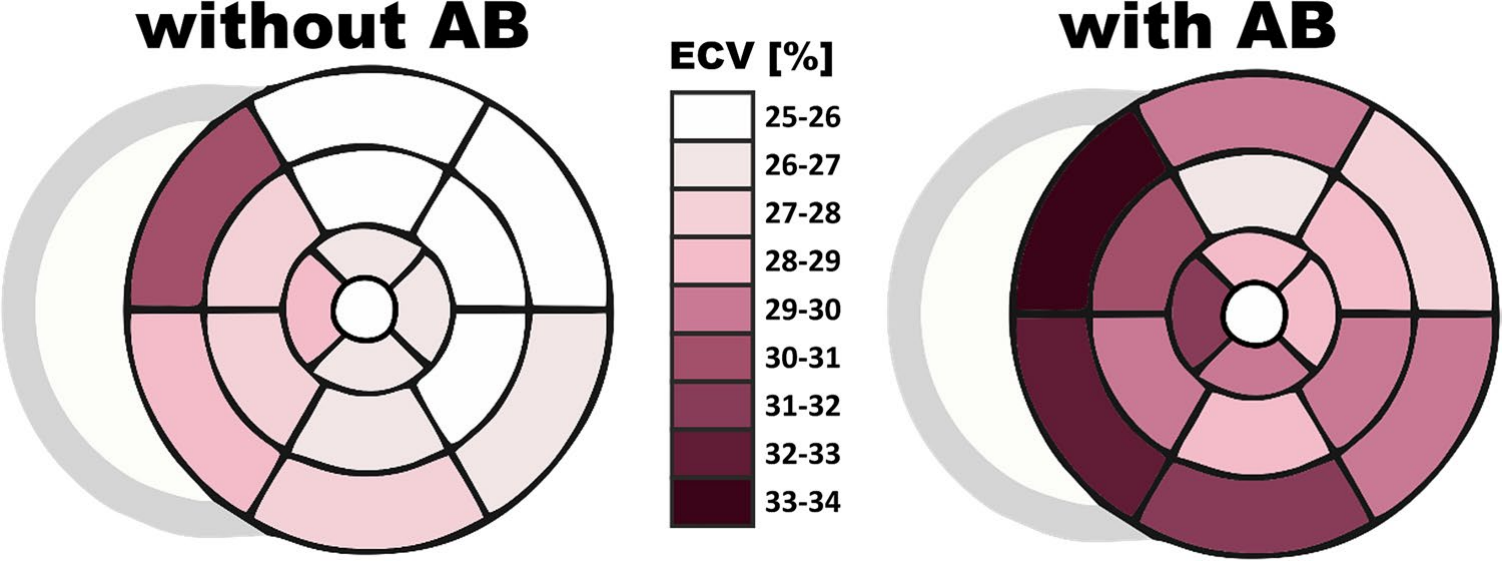
Bull-eye presentation of median extracellular volume (ECV) in dilated cardiomyopathy (DCM) patients with (3A) and without (3B) arrhythmic burden (AB). In both groups, the most fibrotic segments were identified in the septum.

LGE presence and extent were similarly distributed between the groups (Table 2). There were no differences between native T1 times measured at the septum or the average from all segments. There were also no differences between the two groups in native and post-contrast blood T1 times. However, post-contrast T1 times, both at the septum and the average were significantly lower in patients with AB. Similarly, ECV in nearly all myocardial segments, including the septum as well as the global ECV were significantly larger in patients with AB (Table 2, Supplementary Table 2, Fig. 1). Except for the native blood T1 times, which were similar between DCM patients and age- and sex-matched controls, all other T1 parametric measures differed between the two groups (Supplementary Table 1).

**Table 2:** Comparison of ECG and CMR findings between DCM patients with and without AB.

Parameters	without AB (n = 58)	with AB (n = 41)	*p* value
**ECG**			
QRS (ms)	102.6 ± 30.4	97.1 ± 21.2	0.76
QTc (ms)	428.03 ± 36.58	418.88 ± 61.25	0.97
Mean heart rate (bpm)	70.17 ± 9.99	67.85 ± 8.76	0.35
Arrhythmias			
PAC (/h)	9.42 ± 34.80	33.41 ± 118.72	**0.01**
PVC (/h)	3.40 ± 5.57	123.13 ± 199.61	**< 0.0001**
VT (/24 h)	0	4.56 ± 12.72	**< 0.0001**
Conduction blocks			
SAB (n, %)	2 (3.5)	0 (0.0)	0.23
AVB (n, %)	7 (12.1)	4 (9.8)	0.72
IVB (n, %)	13 (22.4)	5 (12.2)	0.19
Pause (n, %)	2 (3.5)	2 (4.9)	0.72
**CMR**			
LVEF (%)	32.57 ± 10.56	31.45 ± 9.61	0.61
LV mass (g)	186.09 ± 52.77	181.17 ± 49.91	0.66
RVEF (%)	41.63 ± 11.53	40.03 ± 10.29	0.47
LAA (cm^2^)	27.06 ± 8.21	28.59 ± 7.38	0.31
RAA (cm^2^)	25.94 ± 7.36	24.67 ± 5.51	0.56
LGE (n, %)	29 (50.0)	22 (53.7)	0.79
%LGE (%)	4.36 ± 5.77	4.68 ± 3.98	0.27
T1 native blood (ms)	1791.38 ± 259.77	1785.05 ± 183.69	0.66
T1 post-contrast blood (ms)	315.77 ± 45.13	312.58 ± 48.65	0.76
T1 native septal (ms)	1274.39 ± 92.37	1245.79 ± 213.47	0.30
T1 native global (ms)	1241.67 ± 88.77	1239.49 ± 216.6	0.06
**T1 post-contrast septal **(**ms**)	**476.25 ± 52.28**	**441.01 ± 45.01**	**0.002**
**T1 post-contrast global **(**ms**)	**484.36 ± 49.59**	**454.90 ± 40.10**	**0.005**
**ECV septal **(**%**)	**29.16 ± 5.37**	**32.08 ± 5.27**	**0.02**
**ECV global **(**%**)	**27.94 ± 4.99**	**30.34 ± 4.22**	**0.02**

Values are mean ± SD or n (%).

*AB* arrhythmic burden, *ECG* electrocardiogram, *QTc* corrected QT, *PVC/PAC* premature ventricular/atrial contractions, *24 h/h* 24 h/hour, *VT* (non-sustained or sustained) ventricular tachyarrhythmia, *SAB* sinoatrial block, *AVB* atrioventricular block, *IVB* intraventricular block, *pause* pause > 3 s, *CMR* cardiovascular magnetic resonance, *LV/LVEF* left ventricle /LV ejection fraction, *LAA/RAA* left/right atrial area, *LGE* late gadolinium enhancement, *%LGE* extent of LGE, *ECV* extracellular volume, *septal* mean value of 8- and 9-segment.

### Associations between ECV and LVEDd with AB in DCM

**Figure 2:**
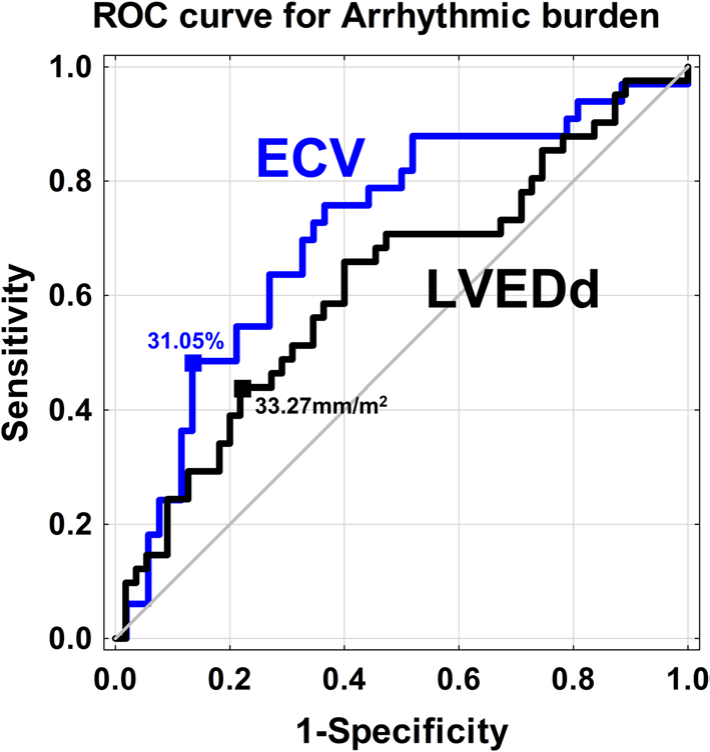
ROC curve of extracellular volume (ECV) and indexed left ventricle end-diastolic diameter (LVEDd) with cut-off points for the presence of arrhythmic burden.

Out of all the parameters that differentiated patients with and without AB (with *p* < 0.10), only indexed LVEDd and the global ECV were found to be independently associated with AB (Table 3). The ECV had a higher diagnostic yield (*p* = 0.04) of discriminating AB presence (AUC 0.713, 95%CI 0.598–0.827; *p* < 0.001) than indexed LVEDd (AUC 0.62, 95%CI 0.504–0.735; *p* = 0.04) The proposed cut-off point of ECV of 31.05% had a higher specificity and sensitivity than indexed LVEDd cut-off point of 33.27 mm/m^2^ (sensitivity/specificity: 55%/79% vs. 44%/78%, respectively). The proposed cut-off points of ECV and indexed LVEDd had the highest accuracy (72% vs. 64%, respectively) (Fig. 2). An increase in ECV by 1% increases the risk of AB occurrence by 12%. ECV > 31.05% increased AB sixfold (HR 6.19; 95%CI 1.95–19.63; *p* = 0.002) with adjustment for the presence of hypertension, systolic blood pressure, LV end-diastolic indexed diameter and loop diuretics’ daily dosage.

**Table 3:** Uni- and multivariate regression models for arrhythmic burden presence.

Parameters	Univariate		Multivariate	
	OR [95%CI]	*p* value	OR [95%CI]	*p* value
Hypertension (n, %)	0.36 [0.12–1.10]	0.07	–	–
SBP (mmHg)	0.98 [0.96–1.00]	0.09	–	–
**LVEDd/BSA **(**mm/m**^**2**^)	**1.10 [1.00–1.21]**	**0.04**	**1.10 [1.00–1.22]**	**0.04**
Creatinine (umol/l)	0.99 [0.96–1.01]	0.2	–	–
Loop diuretics dosage (mg/24 h)	0.99 [0.99–1.01]	0.60	–	–
**ECV global **(**%**)	**1.12 [1.01–1.25]**	**0.04**	**1.12 [1.00–1.25]**	**0.04**

*OR* odds ratio; *CI* confidence interval; other abbreviations as in Tables 1 and 2.

### Reproducibility of measurements

The reproducibility of native and post-contrast T1 was tested in 31 randomly selected subjects and resulted in a coefficient of variability for the intra-observer T1 measurement of 0.77% and 1.22% (bias: − 5.89 ms and − 8.31 ms, respectively) and for the inter-observer T1 measurements of 1.02% and 2.34% (bias: − 0.27 ms and − 0.86 ms, respectively). The Bland–Altman curves are presented in the Supplementary Fig. 1.

## Discussion

The study findings can be summarized as follows. Patients with and without AB were indistinguishable according to numerous demographic, clinical, echocardiographic and laboratory parameters as well as baseline therapy with the exception of larger LV cavities in DCM patients with AB. Neither qualitative nor quantitative LGE-based assessment of replacement fibrosis allowed for the stratification into low or high AB. ECV in nearly all myocardial segments as well as the global ECV were larger in patients with AB. ECV and indexed LV end-diastolic diameter were independently associated with AB in DCM.

### Arrhythmic risk stratification in DCM

Accurate arrhythmic risk stratification is a cornerstone of optimal management of the DCM patients. However, current guidelines select DCM patients for implantable cardioverter-defibrillator (ICD) implantation for primary prevention of SCD based on only three parameters: symptoms (according to NYHA class), persistent LVEF ≤ 35% despite of at least 3 months of optimal medical therapy and an expected survival longer than 1 year^12–14^. In practice, LVEF remains the cornerstone of risk-stratification. However, LVEF has a low sensitivity and specificity for the prediction of SCD^18^. The DANISH study revealed that ICD implantation did not result in a reduction of SCD but was associated with an increased rate of device-related adverse events in DCM^19^. In the landmark meta-analysis based on 45 studies enrolling 6,088 patients, Goldberger et al. evaluated the predictive power of 12 commonly used arrhythmic risk tools. The authors reported prognostic accuracies with a range of sensitivities (28.8–91%) and specificities (36.2–87.1%) for all studied tests with the best performance for fragmented QRS and T-wave alternans^20^. In this study, the prevalence of NSVT was 45.5%, and it had moderate diagnostic accuracy for SCD prediction (sensitivity 64%, specificity 57.5%, and OR 2.92)^20^.

DCM is commonly diagnosed in young adults in whom it is the primary reason for HF and SCD^2,13,14^. However, in almost all contemporary DCM-dedicated studies on SCD, enrolled patients are in their sixth or even seventh decade, e.g. Dawson et al.—mean age of 48–57 years, aus dem Siepen et al.—55–58 years, Nakamori et al.—56 ± 16 years, Cui et al.—49.3 ± 16, Køber et al.—63–64 years, Goldberger et al.—52.8 ± 14.5 years, Nakamori et al.—52 years (range 19–89), Neilan et al.—55 years, Kodama et al.—61 ± 12 years^2,9–11,19–23^. Our study is one of the very few in which the mean age of DCM patients is much younger (45.2 ± 11.8 years) and more likely reflects the true spectrum of DCM population. In addition, our study population of almost 100 DCM patients with T1-parametric assessment is one of the largest published cohorts as the majority of studies enrolled a smaller number of patients. Of note, all data on the associations between investigated parameters and outcomes should be interpreted with caution given the size of the studies. Thus, we focused on the established predictor of arrhythmic events—arrhythmic burden.

### Replacement fibrosis

Several studies have reported an association between LGE and ventricular arrhythmias and SCD^2,15,16^. However, the majority of those studies stratified patients based on a binary classification into: LGE-present and LGE-absent groups, which is an oversimplification and gross underestimation of the true burden of cardiac fibrosis. There are several studies that quantified the extent of LGE in the context of arrhythmic risk. In one study, the amount of cardiac fibrosis, expressed as LGE extent, was found to be an independent SCD predictor. Neilan et al. reported that LGE extent was the strongest predictor of major events, including SCD and appropriate ICD therapy, and patients with LGE involving > 6.1% of LV had the highest rate of events^22^. It should be emphasized that LGE extent is variably measured in studies, and there is still no consensus on the best method of quantification^24^. In our study LGE was present in 41% of patients and LGE extent constituted approximately 4.5% of the LV. Both qualitative (LGE “+” or LGE “−”) and quantitative (LGE extent) assessments showed very similar LGE distribution in patients with and without AB. Consequently, neither LGE presence nor LGE extent were found to be associated with AB.

### Interstitial fibrosis

CMR-based parametric mapping overcomes numerous shortcomings of LGE-based assessment of cardiac fibrosis. Crucially, T1-mapping allows for the quantification of interstitial fibrosis. In order to ensure high quality and reproducible measurements, acquired parametric data should be compared with: (1) published referral values, and (2) an internal control group. An impressive number of 5541 healthy subjects with CMR mapping were aggregated in a recent meta-analysis, which showed a mean native T1 time of 1159 ms (95%CI 1143–1175 ms) at 3.0-T for MOLLI-based protocol^25^. Based on measurements in 3872 volunteers, normal values for ECV have been established as 25.9% (95%CI 25.4–26.5%) at 3.0-T^25^. Our examinations were performed at 3.0-T utilizing the MOLLI protocol; mean native T1 times in the control group were 1196.3 ± 34.8 ms in comparison to DCM patients in whom an average native T1 time was 1232.2 ± 174.7 ms. The average ECV in the control group was 23.9 ± 1.2% and the septal ECV was 26.3 ± 3.5%, whereas in our patients it was 28.7 ± 4.9% and 32.2 ± 7.7%, respectively. We also report very high reproducibility of measurements, both for intra- and inter-variability assessments. Thus, we believe our data represents high-quality measurements.

Since 2013, a few dozen studies utilizing CMR T1 mapping in DCM have been published. Native and post-contrast T1 times as well as ECV have been found to be associated with LV reverse remodelling and outcomes in DCM. There are only a few studies that have investigated the relationships between T1 parameters and ventricular arrhythmias in DCM^17,21,23^. Nakamori et al. compared 50 DCM patients with a history of complex ventricular arrhythmias (ComVA) with 57 age-matched DCM patients without ComVA. The authors found that global native T1 time was significantly higher in patients with ComVA in comparison to those without ComVA (1131 ± 42 vs. 1107 ± 45 ms). Moreover, native T1 was found to be independently associated with ComVA, even after adjusting for LV function and LGE^17^. In the another paper, Nakamori et al. examined 115 DCM patients referred for SCD primary prevention assessment. The combination of native T1 time and the novel index—Mad-SD (which is an index of mean absolute deviation of the segmental pixel-SD from the average pixel-SD) showed excellent diagnostic accuracy in regard to the prediction of arrhythmic event-free survival^21^. Kodama et al., who studied 60 DCM patients, showed that patients with ECV ≥ 30% and QRS duration > 120 ms had a higher rate of arrhythmic events^23^. In the present study, we measured ECV in 16 myocardial segments, which allowed for the calculation of global ECV as well as an average of two mid-myocardial septal segments (no. 8 and 9 according to AHA classification). We observed that septal segments, regardless of anatomical location—basal, mid-ventricular or apical, had the highest ECV values, which is consistent with previous reports. This ECV gradient is common in patients with and without AB; however, all septal ECV values were significantly higher in those with AB.

Until recently, it was not feasible to reliably assess and quantify interstitial fibrosis. The advent of the CMR parametric technique has dramatically changed this perspective. Nonetheless, it should be acknowledged that ECV is not a 100% specific measure of fibrosis given that other interstitial pathologies, such as oedema, protein degradation and aggregation, lipid accumulation, and deposition of iron or amyloid profoundly impact on ECV values^5–7^. These aforementioned conditions are infrequent and have been excluded such that ECV is believed to reflect interstitial fibrosis. It is not surprising that wide-spread interstitial fibrosis negatively affects electrical signal transmission between cardiomyocytes and may promote the development of arrhythmias. Interstitial fibrosis (i.e. increased ECV) should not be viewed as a direct mechanism of arrhythmia in a particular patient as those mechanisms are much more complex and include scar related re-entry circuit, bundle branch block re-entry or mutations of ion channels^14^. Thus, ECV (i.e. increased ECV) should be considered a very strong marker of ventricular arrhythmias, specifically in DCM.

It may be time to challenge the paradigm of cardiac fibrosis in the setting of DCM. A binary classification scheme of cardiac fibrosis seems to be outdated in the era of quantitative assessment of fibrosis. Cardiac fibrosis, as almost all biological processes, is a continuum, and it is a gross assessment to dichotomize between absent and present as “present” may infer significantly different degrees of fibrosis. In the early days of CMR, when only LGE-based assessment was present, it may have been a reasonable description of results. However, in the era of quantitative assessment of fibrosis, the “degree” of cardiac fibrosis may be precisely defined. Based on ROC analysis, we found that ECV > 31% accurately stratifies patients into low and high arrhythmic risk. Cardiac fibrosis is present in both sub-groups, as ECV is already elevated in all of them (in comparison to the controls); however, it is the amount of fibrosis that increases the arrhythmic risk.

### Study limitations

Although our study is one of the largest published studies, it is a single-centre study with a relatively small number of patients. Despite the fact that CMR T1 parametric mapping is already a mature technique, several different imaging protocols exist. We relied on the most commonly-used MOLLI protocol; however, utilizing other protocols may provide slightly different results. Due to the lack of complete data from historic reports or examinations, AB was based on a 48-h Holter monitor during the index hospitalization or outpatient visit.

## Conclusions

Neither qualitative nor quantitative LGE-based assessment of replacement fibrosis allowed for the stratification of DCM patients into low or high AB. Interstitial fibrosis, expressed as ECV, was significantly higher in patients with AB. ECV alongside with indexed LV diameter were found to be the only independent predictors of AB in DCM. Incorporation of CMR parametric indices into predictive models may improve arrhythmic risk stratification in DCM.

## Methods

### Study population

Between May 2019 and September 2020, 102 consecutive patients with a diagnosis of DCM were recruited. All patients received optimal heart failure (HF) therapy and displayed stable symptoms (New York Heart Association—NYHA I–III class) for at least 2 weeks prior to enrollment. DCM was diagnosed based on (1) the presence of impaired left ventricle (LV) systolic function (ejection fraction, EF < 45%) and LV dilation, and (2) the exclusion of significant coronary artery disease (CAD, > 50% luminal stenosis), primary heart valve disease, congenital heart disease and severe arterial hypertension^26^. Our control group consisted of 11 healthy volunteers who were free of any chronic diseases.

### Study design

This is a prospective, single-centre observational study. All patients underwent detailed diagnostic work-up, including clinical evaluation, laboratory tests, electrocardiogram (ECG), 48-h Holter ECG monitoring, transthoracic echocardiography, 6-min walk test (6MWT) and CMR. The patients were on guideline-directed medical therapy for HF including beta-blockers (BB), angiotensin receptor-neprilysin inhibitor (ARNI) or angiotensin-converting-enzyme inhibitor (ACE-I), mineralocorticoid receptor antagonist (MRA) and loop diuretics^12^. The study protocol was approved by the John Paul II Institutional Review Board and the Krakow Medical Chamber Ethics Committee (reference number 7/KBL/OIL/2019). All study-related procedures were performed in accordance with the current Guideline for Good Clinical Practice and Declaration of Helsinki 2013. All patients and volunteers gave written informed consent.

### Cardiac magnetic resonance

CMR examinations were performed at the time of the inclusion on a 3.0-T scanner (Magnetom Skyra, Siemens, Erlangen, Germany). CMR studies were analysed by using Syngo.VIA software version VB 40 (Siemens, Erlangen, Germany) following the post-processing guideline from the Society of Cardiovascular Magnetic Resonance^7,8^. Steady-state free precession cine images were obtained in consecutive short-axis slices covering the LV and three long-axis (2-, 3-, and 4-chamber) slices. The CMR protocol included cine CMR, native and post-contrast T1 mapping, and LGE imaging. The LV was divided into 16-segment model described by the American Heart Association. Cardiac volumetric and functional parameters were quantified based on manual delineation of the endocardial and epicardial borders using a stack of continuous short-axis slice cine images.

### Assessment of replacement fibrosis

**Figure 3:**
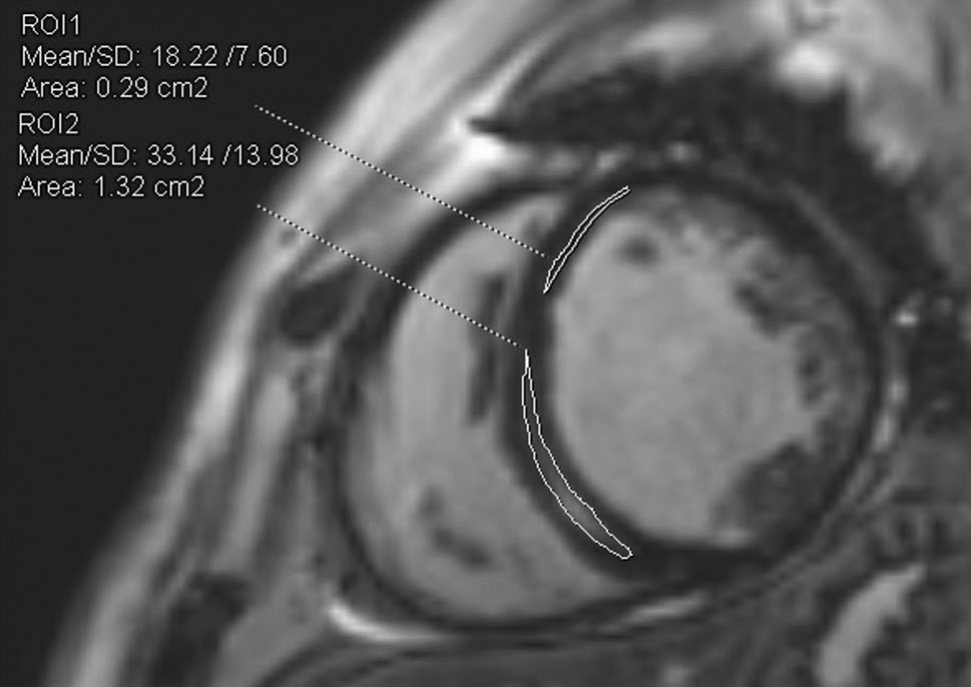
Late gadolinium enhancement (LGE) images demonstrating quantification of the septal burden of replacement fibrosis (encircled) using the 3 standard deviations threshold on consecutive short-axis slices technique.

Consecutive short-axis LGE images covering the LV were obtained 15 min after intravenous injection of 0.1 mmol/kg of body weight gadolinium-based contrast agent. The presence of LGE was judged by three independent observers blinded to patients’ information (M.U., M.K. and M.M.). Fibrosis was considered present if LGE was visualized on both short- and orthogonal long-axis LGE images. The quantitative analysis of LGE extent was assessed using 5 standard deviations threshold on consecutive short-axis slices and calculated as a percentage of total LV mass (%LGE) (Fig. 3)^7,8^.

### Assessment of interstitial fibrosis

**Figure 4:**
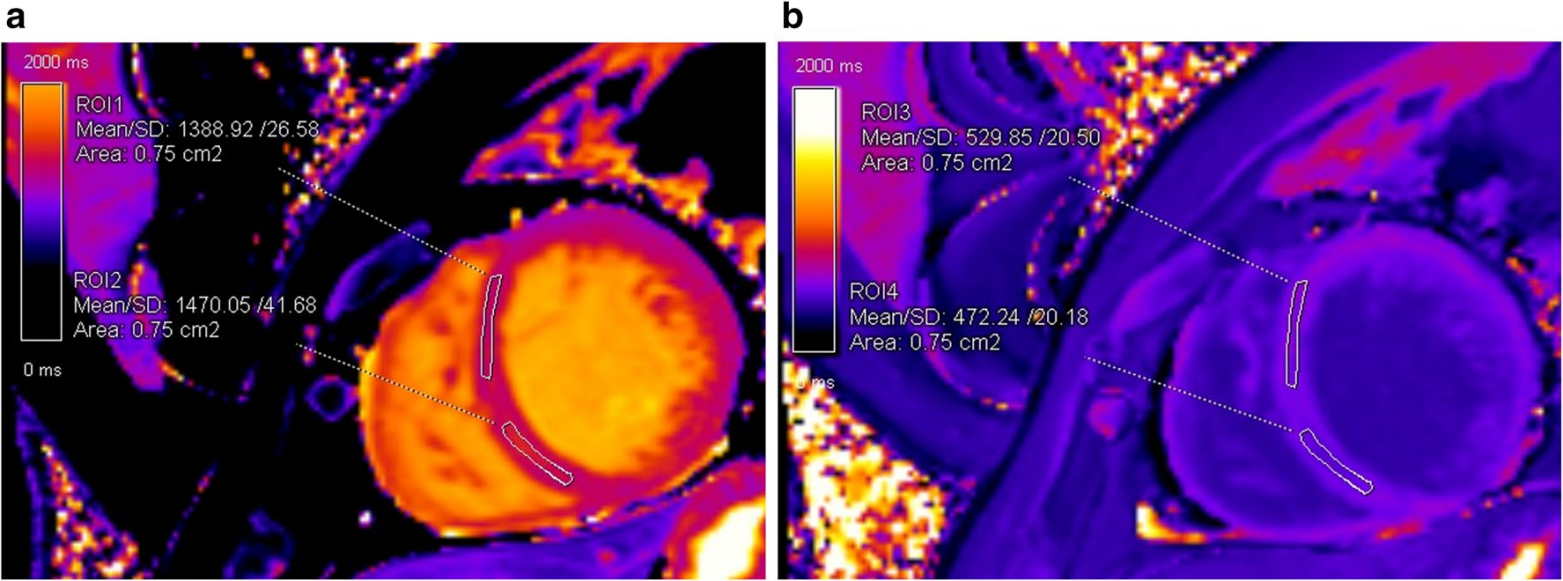
Native and post-contrast contours in the mid-myocardial area. The left ventricular cavity is shown (orange in native T1-mapping and dark-blue in post-contrast T1-mapping) to enable derivation of blood and myocardial T1 values. Standardized ROIs are placed in the septum to measure native (**A**) and post-contrast T1-times (**B**).

T1 mapping was performed using (Siemens Skyra VE11 with MyoMaps) a Modified Look Locker Inversion (MOLLI) Recovery sequence before and 15 min after gadolinium-based contrast agent injection. The following typical MOLLI sequence tfi2Dl parameters were used: breath-hold TR/TE of 281/1.1 ms, slice thickness of 8 mm, FOV from 320 × 260 mm^2^, matrix of 144 × 256 pixels, flip angle of 35°. Native and post-contrast T1-values were determined by drawing regions of interest (ROI) in every segment of the basal, mid-ventricular slice as well as in the centre of LV cavity for measuring T1 blood pools. ROIs were drawn in mid-wall region of the myocardial segments and were copied between native and post-contrast T1 maps (Fig. 4A,B). Segments with artifacts were excluded. The global native, post-contrast T1 times as was well as ECV were calculated as a mean of all segments. Septal ECVs were calculated as the mean of mid-myocardial 8 and 9 segment (assessment blinded to the clinical data, P.B). ECV was calculated according to the established formula:

$$ ECV = \left( {{{\frac{1}{postcontrast T1} - \frac{1}{native T1}} \mathord{\left/ {\vphantom {{\frac{1}{postcontrast T1} - \frac{1}{native T1}} {\frac{1}{blood postcontrast T1} - \frac{1}{blood native T1}}}} \right. \kern-\nulldelimiterspace} {\frac{1}{blood postcontrast T1} - \frac{1}{blood native T1}}}} \right){*}\left( {1 - {\text{Hct}}} \right), $$

Areas of LGE replacement fibrosis were included in the T1 times and ECV measurements^7,8^. A blood sample was obtained on the day of scanning to measure hematocrit.

### Electrocardiographic examinations

Patients underwent 48-h ECG Holter monitoring (Spacelabs Healthcare, Reynolds Medical, Lifecard CF, United States). The mean, minimum and maximum heart rate, the total number of PVCs and NSVT were recorded. VT was defined as a minimum of three consecutive PVCs with a rate of ≥ 100 bpm. All recorded VTs were non-sustained. We divided the total number of PVCs during the recording by the number of analysis hours (PVC/h) and calculated the daily VT numbers by dividing the total VT number by the 2-days monitoring (VT/d). The AB was defined as the presence of VT or a high burden of PVC (≥ 30 PVCs per hour)^14,27,28^.

### Statistical analysis

All values are presented as mean ± SD or percentage where appropriate. All quantitative variables were tested for the normal distribution of data with the Shapiro–Wilk test. Comparisons of the continuous variables were conducted with a t-test when normality was confirmed, otherwise with a Mann–Whitney test. The χ^2^ test was performed for the comparison of qualitative parameters. The associations between the parameters and the AB were analysed with uni- and multivariate logistic regression methods. All parameters (presented in the Table 1) differentiating AB present versus absent groups (with *p* value < 0.10) were included in the regression analyses. When normality was confirmed, a Pearson correlation was performed in order to identify a potential correlation between AB predictors. Otherwise, a Spearman rank correlation was used. Due to the high correlation between ECV and native and post-contrast T1 times (R-Spearman > 0.8) and between systolic and diastolic blood pressure (SBP and DBP; R = 0.59, *p* < 0.001), only total ECV and SBP were included in the regression models. Areas under (AUC) the receiver operating curve (ROC) were calculated to assess the cut-off values of ECV for AB. The Z test was used for comparing the AUC of each prognostic scale. Agreement between measurements was assessed using Bland–Altman plots. All results were considered statistically significant when the *p* value was < 0.05. The Statistica package, version 13.0 (StatSoft, TIBCO Software Inc.), was used.

## Supplementary Information

Supplementary Information.
