# Supplementary material for: Extracellular volume is an independent predictor of arrhythmic burden in dilated cardiomyopathy
Source: Sci Rep. 2021 Dec 14;11:24000. doi: 10.1038/s41598-021-03452-z (PMC8671445; doi:10.1038/s41598-021-03452-z)

**Supplementary Table 1.** Comparison of baseline characteristics, including CMR parameters between DCM patients and control group.

| **Parameter** | **DCM patients (n=99)** | **control group (n=11)** | **p-value** |
| --- | --- | --- | --- |
| Age [years] | 45.23 ± 11.76 | 40.89 ± 12.27 | 0.24 |
| Male sex (n, %] | 89 (87.3) | 6 (66.7) | 0.09 |
| **BMI [kg/m^2^]** | **28.49 ± 5.68** | **24.83 ± 3.75** | **0.05** |
| **LVEDd [mm]** | **63.81 ± 8.19** | **45.00 ± 7.24** | **0.0005** |
| **LVEF [%]** | **29.66 ± 10.02** | **58.60 ± 2.51** | **0.0002** |
| **T1 native septal [ms]** | **1285.1 ± 163.8** | **1236.9 ± 51.3** | **0.001** |
| **T1 native global [ms]** | **1232.2 ± 174.7** | **1196.3 ± 34.8** | **0.003** |
| T1 native blood [ms] | 1793.5 ± 230.8 | 1849.5 ± 63.7 | 0.58 |
| **T1 post-contrast septal [ms]** | **440.9 ± 75.7** | **637 ± 34.4** | **<0.001** |
| **T1 post-contrast global [ms]** | **470.9 ± 49.1** | **631.4 ± 38.5** | **<0.001** |
| **T1 post-contrast blood [ms]** | **311.8 ± 48.5** | **458.8 ± 51.9** | **<0.001** |
| **ECV septal [%]** | **32.2 ± 7.7** | **26.3 ± 3.5** | **0.01** |
| **ECV global [%]** | **28.7 ± 4.9** | **23.9 ± 1.2** | **0.002** |

Values are mean ± SD or n (%).

Abbreviations: BMI – body mass index, LVEDd – left ventricle end-diastolic diameter, LVEF – left ventricle ejection fraction, ECV – extracellular volume.

**Supplementary Table 2**. Comparison of median value (25^th^ percentile, 75^th^ percentile) of extracellular volume (ECV) of each segment between DCM patients with and without arrhythmic burden (AB).

| **ECV [%]** | **with AB (n=41)** | **without AB (n=58)** | **p-value** |
| --- | --- | --- | --- |
| **Segment 1** | 29.28 (24.25; 32.69) | 25.24 (23.33; 28.18) | **0.009** |
| **Segment 2** | 33.85 (29.37; 37.14) | 30.57 (27.14; 33.84) | **0.04** |
| **Segment 3** | 32.66 (27.8; 37.61) | 28.01 (25.94; 30.98) | **0.004** |
| **Segment 4** | 30.47 (26.69; 33.66) | 27.63 (25.56; 29.21) | **0.02** |
| **Segment 5** | 29.59 (26.9; 33.42) | 26.24 (24.44; 30.17) | **0.04** |
| **Segment 6** | 27.45 (24.75; 32.44) | 25.26 (23.75; 27.85) | **0.03** |
| **Segment 7** | 26.43 (24.52; 30.62) | 25.42 (23.42; 27.8) | 0.158 |
| **Segment 8** | 30.35 (27.98; 33.38) | 27.3 (24.56; 30.03) | **0.001** |
| **Segment 9** | 29.74 (27.68; 34.71) | 27.36 (24.68; 29.57) | **0.002** |
| **Segment 10** | 28.82 (26.3; 31.35) | 26.46 (24.21; 28.99) | **0.03** |
| **Segment 11** | 29.38 (26.62; 34.13) | 25.85 (23.35; 28.56) | **0.001** |
| **Segment 12** | 28.01 (25.15; 32.33) | 25.65 (24.1; 28.45) | **0.01** |
| **Segment 13** | 28.92 (25.89; 32.49) | 26.46 (23.44; 28.89) | **0.02** |
| **Segment 14** | 31.81 (27.96; 34.68) | 28.03 (25.57; 30.15) | **0.006** |
| **Segment 15** | 29.49 (26.1; 33.06) | 26.16 (23.93; 29.39) | **0.01** |
| **Segment 16** | 28.84 (26.36; 33.72) | 26.25 (24.24; 29.14) | **0.03** |

Values are median (interquartile range).

**Supplementary Figure 1.** Intra- and inter-observer variability of T1-mapping measurements. The reliability and reproducibility of T1 time measurements were assessed by both intra- and inter-observer analyses. (**A-B**) Intra-observer measurements of native (A) and post-contrast (B) T1 times between datasets (Bland–Altman). (**C-D**) Inter-observer measurements of native (C) and post-contrast (D) T1 times between data sets (Bland–Altman).


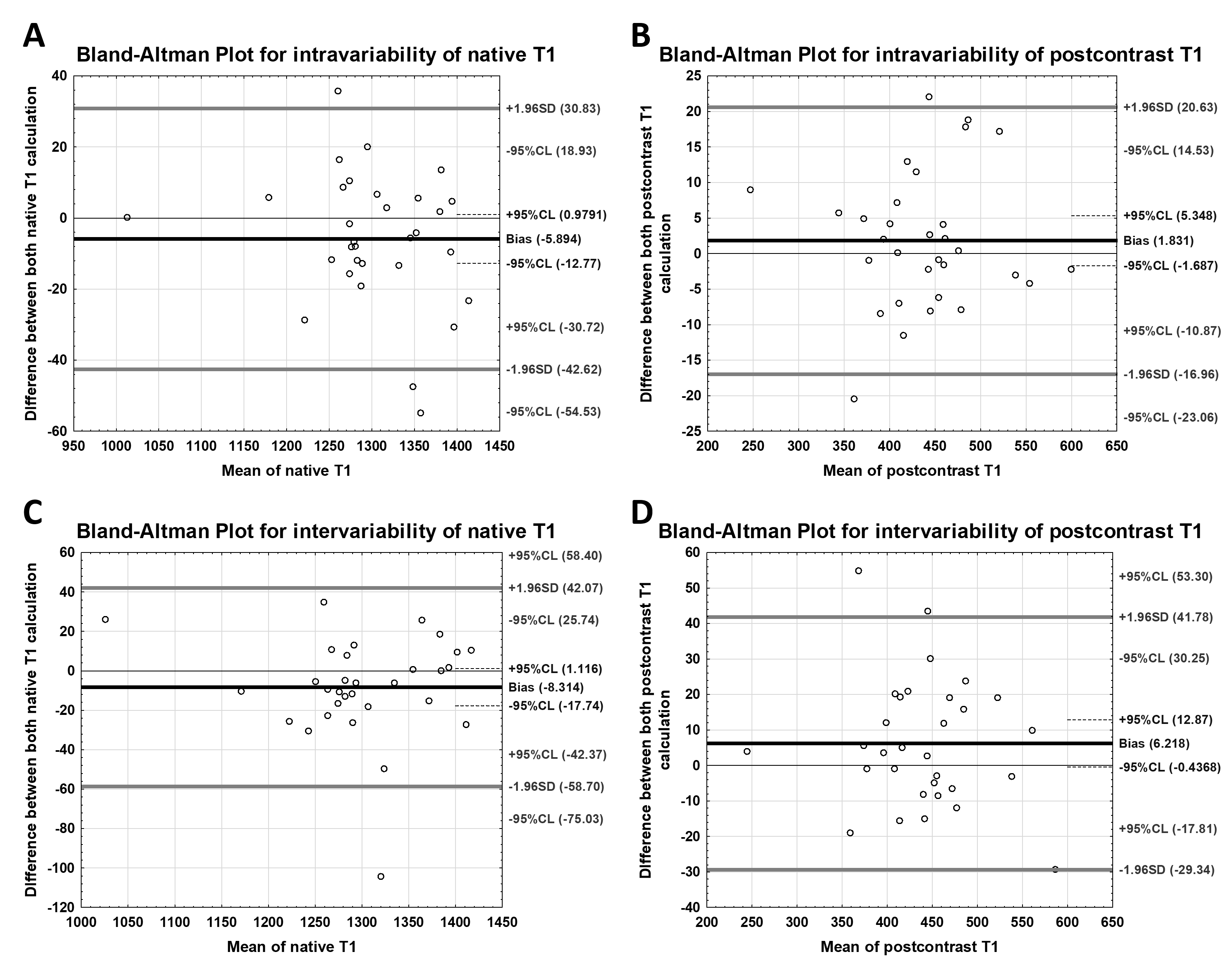

Supplement: Supplementary file 1 — Supplementary Information. [file 41598_2021_3452_MOESM1_ESM.docx]
